# The complete chloroplast genome of *Primula beesiana*, an ornamental alpine plant from SW China

**DOI:** 10.1080/23802359.2019.1698377

**Published:** 2019-12-12

**Authors:** Zhikun Wu, Xiong Chen, Li Zhang, Yuan Huang

**Affiliations:** a Department of Pharmacy, Guizhou University of Traditional Chinese Medicine, Guiyang, P. R. China;; b School of Life Sciences, Yunnan Normal University, Kunming, Yunnan, P. R. China

**Keywords:** Complete chloroplast genome, *Primula beesiana*, perennial alpine species

## Abstract

*Primula beesiana* Forrest is a perennial rosette alpine species in the *Sect. Proliferae* of family Primulaceae. Here, the first complete chloroplast genome of *P. beesiana* was determined. The size of the complete chloroplast genome of *P. beesiana* is 150873 bp with GC content of 37.1%. The assembled genome has a typical quadripartite structure, containing a large single-copy (LSC) region was 82850 bp, a small single-copy (SSC) region was 17,651 bp, and a pair of inverted repeats (IRs) regions was 25,186 bp. There are 137 genes are annotated in the whole cp genome with 91 protein-coding genes, 8 rRNA genes, and 38 transfer RNA (tRNA) genes, including 115 unique genes, 81 unique CDSs, 30 unique tRNAs, and 4 unique rRNAs. The phylogenetic tree showed that *P. beesiana* is closely related to *P. bulleyana.*

*Primula beesiana* Forrest is a perennial rosette alpine species in the Sect. *Proliferae* of family Primulaceae. *Primula beesiana* is structurally very similar to *P. bulleyana* with the color of the flower as the only distinguishing character. The two have natural hybridization and occur in the same area, which narrowly is distributed in alpine meadows and on the sides of streams and ditches in the eastern Himalaya and the Hengduan Mountains (Huang et al. 2015). In particular, *P. beesiana* and *P. bulleyana* play a significant role in ornamental garden hybrids. However, the taxonomic status of *P. beesiana* is uncertain, which was treated as a subspecies of *P. bulleyana* (Richards 1993, 2003), or as an independent taxon by Chinese Plant taxonomist (Hu and Kelso 1996). In this study, we report the complete chloroplast genome sequence of *P. beesiana* for revealing the evolution status of *P. beesiana* based on a phylogenetic tree.

The fresh leaves of *P. beesiana* were provided from Yulong Snow Mountain (Lijiang, Yunnan, China). Voucher specimens (accession no: WZK140715) were deposited in Yunnan Normal University. Total genomic DNA was extracted from the isolated chloroplasts by a modified CTAB method (Sahu et al. 2012). Then, we used Illumina Hiseq X Ten sequencer to construct the genomic library for Illumina paired-end (PE) sequencing and the complete chloroplast genome of *P. beesiana* was assembled using the software NOVOPlasty v2.7.2 (Dierckxsens et al. 2016). The annotation of chloroplast genome assembled was carried out with Geneious v8.0.2 software (Matthew et al. 2012).

The annotated complete chloroplast genome of *P. beesiana* is 150,873 bp in length with GC content of 37.1% (GenBank accession MN504639). The assembled genome has a typical quadripartite structure, containing a large single-copy (LSC) region was 82850 bp, a small single-copy (SSC) region was 17,651 bp, and a pair of inverted repeat (IRs) regions was 25,186 bp. In total, there are 137 genes, 91 protein-coding genes, 8 rRNA genes, and 38 tRNA genes are annotated in the whole cp genome, including 115 unique genes, 81 unique CDSs, 30 unique tRNAs, and 4 unique rRNAs.

To explore the taxonomic status of *P. beesiana* within Sect. *Proliferae* and related species, the phylogenetic relationships were inferred based on the complete chloroplast genomes of 10 species from GenBank, including *P. bulleyana* (MN428416) and 9 related species. These species were aligned by software MAFFT (Katoh and Standley 2013). The maximum likelihood (ML) tree was generated by the software IQ_TREE 1.6.2 (Nguyen et al. 2015) and the branch support and SH-like approximate likelihood ratio (SHAlrt) (Guindon et al. 2010) was estimated with 10,000 bootstrap replicates base on K3Pu + F+I + G4 model according to Bayesian information criterion by the software ModelFinder (Kalyaanamoorthy et al. 2017) (Figure 1). The phylogenetic tree showed that *P. beesiana* is closely related to *P. bulleyana,* forming a monophyletic clade with 100% bootstrap value, which can provide evidence that the two species are very similar in structure. In summary, the complete chloroplast genome sequence of *P. beesiana* can provide essential data for phylogenetic studies of Primulaceae.

**Figure 1.**
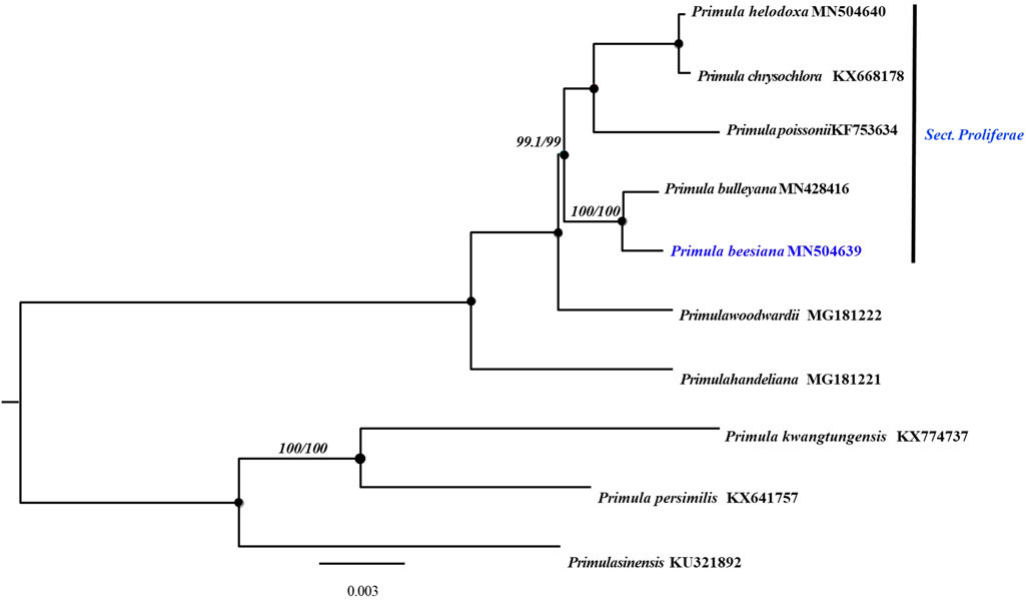
ML phylogenetic tree of *P. beesiana* and 9 Primulaceae species based on chloroplast complete genome, branch support values were reported as SH-aLRT, black solid dot denotes support values of 100/100.
